# Purple Urine Bag Syndrome as a Visual Trigger for Overtreatment: A Case Report and Aggregated Literature Analysis

**DOI:** 10.7759/cureus.101557

**Published:** 2026-01-14

**Authors:** Micah Pippin, Yashwanth Ramineni Damodhar, Sanjay Shrestha

**Affiliations:** 1 Family Medicine, Louisiana State University Health Sciences Center, Alexandria, USA; 2 Family Medicine, Rapides Regional Medical Center, Alexandria, USA

**Keywords:** antibiotic stewardship, asymptomatic bacteriuria, purple urine bag syndrome, urinary catheter, urinary tract infection

## Abstract

Purple urine bag syndrome (PUBS) is an uncommon yet visually striking clinical phenomenon characterized by purple discoloration of urinary drainage systems in patients with indwelling urinary devices. Although alarming, it most often occurs in elderly or chronically ill patients with multiple risk factors for urinary bacterial colonization and does not typically represent a pathologic entity. PUBS is often accompanied by abnormal urine studies, which may prompt antimicrobial treatment even in the absence of clear clinical infection.

We report a hospice patient with an indwelling urinary catheter who developed PUBS without localized urinary symptoms or systemic signs of infection. The patient remained clinically stable, and the treating hospice team did not initiate laboratory evaluation or antibiotic therapy. However, the long-term care facility subsequently administered intravenous ertapenem in response to urine culture results obtained during the episode. The purple discoloration resolved without clinical deterioration.

This case illustrates how PUBS may trigger treatment escalation based on laboratory findings rather than clinical presentation, particularly in vulnerable patients, and was the impetus for a focused examination of management principles described in published reports. Careful interpretation of PUBS within the context of patient symptoms and goals of care may help avoid unnecessary interventions and support more judicious antimicrobial use and antibiotic stewardship.

## Introduction

Purple urine bag syndrome (PUBS) is a conspicuous discoloration of urine collection systems most commonly observed in elderly, catheterized patients. It was first described in the late 20th century and is characterized by purple discoloration secondary to the interaction of tryptophan-derived bacterial metabolites with the plastic components of urinary drainage devices [1-3]. Although its appearance is dramatic, PUBS itself is not inherently pathologic, nor does it signal imminent morbidity [4-5]. Despite this, PUBS is often described in the literature as a concerning indicator of urinary tract infection (UTI) necessitating antimicrobial therapy [4-6]. Many published case reports and reviews frame PUBS as a forerunner of severe infection, referencing the presence of bacteriuria, abnormal urinalysis findings, or apparent resolution following antibiotic treatment as justification for early intervention [4-8]. This assertion persists even in the context of PUBS-affected patient populations where asymptomatic bacteriuria is almost ubiquitous, and treatment is not routinely indicated [9]. While well-documented symptom-based criteria are recommended to diagnose and guide therapeutic decisions for potential UTIs, inappropriate independent variables, such as urine appearance and odor, laboratory findings, and microbiologic culture results, are often used to warrant antibiotic administration [9]. This misperception is especially germane to PUBS, where the visually dramatic finding of purple urine seems to nullify guideline-based reasoning and contribute to reflexive treatment. The extent to which PUBS is associated with symptomatic infection and the frequency with which it leads to unnecessary antibiotic use have not been systematically quantified. To address this gap, and in the context of our own index case, we performed an aggregated analysis of published PUBS cases to examine symptom status, risk factors, management patterns, and outcomes. We sought to assess whether PUBS reliably reflects symptomatic UTI, investigate common treatment practices and the reasoning behind them, and evaluate how these findings relate to the broader conceptualization of asymptomatic bacteriuria and principles of antibiotic stewardship.

## Case presentation

The patient was a 75-year-old female hospice nursing home patient receiving palliative care. She had suffered a significant decline following a hospitalization for status epilepticus. Upon that admission, she was diagnosed with metabolic encephalopathy secondary to a UTI. During her hospital course, she developed seizures and progressed to status epilepticus requiring intubation. Subsequently, she developed bradycardia, which converted to pulseless electrical activity (PEA) and then cardiac arrest. Return of spontaneous circulation was achieved; however, it was apparent that the patient likely incurred a hypoxic brain injury. The patient’s family reflected on her wishes and decided on extubation with comfort measures and general inpatient hospice care. The patient did stabilize and was eligible for return to her nursing home facility to continue hospice care. Her debilitation was significant following this hospitalization, including bedbound status, chronic respiratory failure necessitating supplemental nasal cannula oxygen, dysphagia, impaired communication, and urinary retention requiring an indwelling Foley catheter. She was dependent on others for all activities of daily living, and her palliative performance scale (PPS) was 30% (normal = 100%). Sometime after returning to the nursing home, the patient developed purple discoloration of the urine in the catheter collection system. The patient did not report any dysuria, abdominal pain, flank pain, fever, rigors, or change in mental status. The patient had a past medical history of cerebrovascular accident, chronic kidney disease (CKD) stage three, type 2 diabetes mellitus, hypothyroidism, hypertension, dyslipidemia, chronic constipation, and recurrent UTIs, including culture-confirmed extended-spectrum beta-lactamase (ESBL) *Escherichia coli*. She had been treated repeatedly with varying antibiotic regimens for UTIs throughout her life. Her surgical history was unknown, as was her family history. Her chronic medications included traditional palliative care therapeutics such as as-needed morphine for pain, lorazepam for episodes of agitation or anxiety, and a bowel regimen including polyethylene glycol and bisacodyl rectal suppositories. Her only documented known drug allergy was to losartan; however, the nature of her reaction to the medication was unknown.

Vital sign assessment documented during the evaluation of her purple urine discoloration included a temperature of 97.8°F (36.5°C), a blood pressure of 127/89 mmHg, a heart rate of 86 beats per minute, a respiratory rate of 16 breaths per minute, and an oxygen saturation 97% on 3 L nasal oxygen.

**Figure 1:**
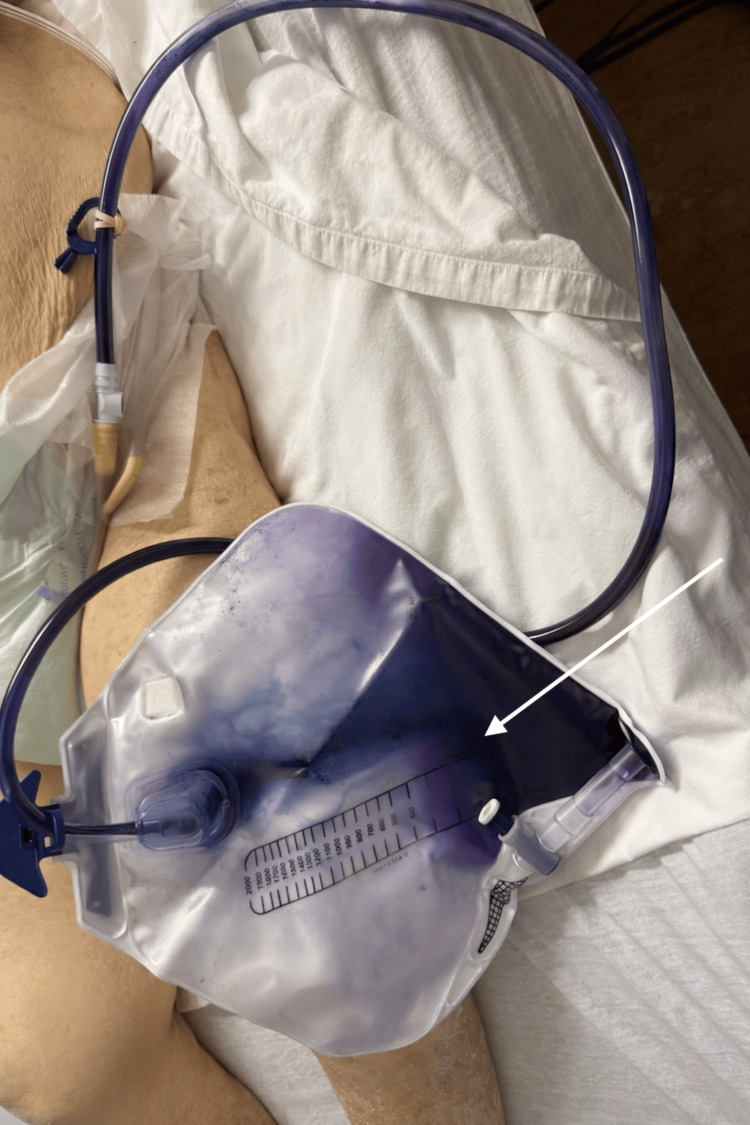
Purple colored urine in catheter tubing and collection bag

The patient’s physical examination was typical of her chronically ill status. She was alert and oriented to person and place but was otherwise confused and disoriented, unchanged from her previous daily evaluations. Her abdomen was soft, nontender, non-distended, with normal bowel sounds. Notably, there was no tenderness in the suprapubic region. Her back examination was normal with no costovertebral angle tenderness to palpation. Her chronic indwelling Foley catheter was present with discolored urine in the collecting bag, appearing purple to deep indigo (Figure 1).

Findings were otherwise benign and unchanged, including cardiac, respiratory, and extremity examinations.

The hospice physician decided to forgo evaluation with urinalysis or culture or initiating empiric antibiotic treatment due to the patient’s asymptomatic presentation and non-contributory physical examination. Two months later, the nursing home practitioner was alerted to the urine discoloration, which was still present, and decided to initiate further evaluation with a dipstick urinalysis and microscopy following catheter exchange. The patient remained asymptomatic with normal vital signs, and her physical examination remained unchanged. The resulting clean-catch urine studies demonstrated a yellow, cloudy sample with moderate blood and a protein of 100 mg/dL. Nitrites were absent, but large amounts of leukocyte esterase were present. Microscopy showed 20-40 red blood cells (RBCs)/high-power field (HPF) and >50 white blood cells (WBCs)/HPF, with associated WBC clumps. There were a few squamous cells present with 4+ bacteria (Table 1).

**Table 1:** Urinalysis laboratory results following catheter exchange

Laboratory	Results	Reference Range
Color	Yellow	Yellow
Appearance	Cloudy	Clear
Glucose	Negative	Negative
Bilirubin	Negative	Negative
Ketone	Negative	Negative
Specific Gravity	1.018	1.002-1.030
Blood	Moderate	Negative
pH	8.0	5.0-8.0
Protein	100 mg/dL	Negative
Urobilinogen	Negative	Negative
Nitrite	Negative	Negative
Leukocyte Esterase	Large	Negative
Red Blood Cells (RBCs)	20-40/HPF	0-3/HPF
White Blood Cells (WBCs)	>50/HPF	0-8/HPF
WBC Clumps	Many/HPF	None/HPF
Squamous Cells	Few/HPF	None/HPF
Bacteria	4+/HPF	None/HPF
Mucous	Trace	None-Trace

In response to the urinalysis findings, the nursing home facility ordered empiric antibiotic coverage with cefdinir 300 mg twice daily, and urine cultures were obtained. The cultures and resulting sensitivities demonstrated ESBL *Escherichia coli* at high concentrations and *Proteus mirabilis* and *Providencia stuartii* at lower concentrations (Table 2).

**Table 2:** Urine culture results and sensitivities following catheter exchange ESBL, extended-spectrum beta-lactamase

Bacteria	ESBL *Escherichia coli*	Proteus mirabilis	Providencia stuartii
Concentration	>100,000 CFU/mL	>10,000 but <50,000 CFU/mL	>10,000 but <50,000 CFU/mL
Amoxicillin/Clavulanic Acid	Sensitive	Sensitive	-
Ampicillin	Resistant	Resistant	-
Cefazolin	Resistant	Resistant	-
Cefepime	Resistant	Resistant	Resistant
Ceftriaxone	Resistant	Resistant	Resistant
Ertapenem	Sensitive	Sensitive	Sensitive
Gentamicin	Sensitive	Sensitive	-
Meropenem	Sensitive	Sensitive	-
Nitrofurantoin	Sensitive	-	-
Tetracycline	Sensitive	-	-
Trimethoprim/Sulfamethoxazole	Resistant	Resistant	-

The patient was moved to a different room and placed on isolation precautions due to ESBL bacteriuria, and intramuscular (IM) ertapenem 1 g daily was initiated. The patient completed the course of antibiotics, and the purple urine discoloration resolved. Of note, the patient's asymptomatic PUBS did recur one month later, and the patient was managed once again with a course of IM carbapenem.

## Discussion

PUBS is an uncommon but striking clinical entity that predominantly occurs in elderly institutionalized and chronically catheterized patients and has a strong female predominance [8-10]. The phenomenon results from bacterial metabolism of dietary tryptophan into indoxyl sulfate, which subsequently converts to the indigo and indirubin pigments in alkaline urine [2-3]. Absorption of these pigments into the polyvinyl chloride components of urinary catheter tubing and collection bags results in the characteristic purple discoloration [3]. The bacterial pathogens most frequently reported to produce PUBS-related enzymes, and the associated syndrome, include *Providencia* and *Proteus* species [10-11]. *Morganella* and *Klebsiella* are also often implicated in the development of PUBS, but with a more moderate frequency [11]. While *Escherichia coli* is commonly isolated from urine cultures collected from patients with PUBS, not all strains produce the necessary enzymes, and its association with the disorder is weaker than that of previously described organisms [11]. ESBL status does not confer any increased risk for pigment formation [11].

Established risk factors for PUBS include indwelling urinary catheters, female sex, age ≥ 70, the bedridden and institutionalized, constipation, CKD, diabetes mellitus, and structural urologic abnormalities [9-11]. The evaluation of PUBS should include a thorough history and physical examination, with particular focus on assessing for urinary and systemic symptoms consistent with a UTI. This investigation is especially prudent as urinalysis and urine cultures often demonstrate pyuria and bacteriuria without true infection, and management of PUBS hinges on the presence or absence of symptom-based criteria for UTI [7-10]. Treatment principles consist of catheter care, addressing predisposing factors such as constipation, and reserving antibiotic therapy for patients with UTI-specific symptoms defined by the Infectious Disease Society of America (IDSA) [9] as including, dysuria, frequency, urgency, suprapubic pain or tenderness, flank pain, costovertebral angle tenderness, fever (≥ 38°C/100.4°F), chills, rigors, sepsis or septic shock, and acute hemodynamic instability [4,7-9]. The differential diagnosis includes hematuria, porphyria, medication effects, or chemical staining, all of which can cause discoloration of the urine and the urine collection system [9-10]. Although some reports have associated PUBS with sepsis or critical illness, the condition is generally benign with favorable outcomes and no inherent association with adverse prognosis [4,9-10]. Prevention centers on minimizing unnecessary urinary catheterization, optimizing catheter hygiene, and avoiding treatment of asymptomatic bacteriuria [9-11].

Our index case is representative of the typical PUBS presentation and illustrates several themes and questions present in today’s PUBS literature, particularly concerning the overall significance of the syndrome, its association with UTIs, and the use of antibiotics in its management. Our patient exhibited almost all the established risk factors for PUBS, including an indwelling catheter, female sex, age, CKD, diabetes mellitus, institutionalization, constipation, and bedbound status [9-11]. Interestingly, laboratory analysis reported the urine color as yellow, a surprisingly common finding in freshly collected and intravesicular urine samples in PUBS. The purple discoloration requires contact with catheter tubing and collection bags, indicating an extracorporeal chemical reaction, rather than an inherent pathologic property of the urine [3]. Our patient’s microbiologic findings also provide a curious insight into the nature of PUBS and management philosophy. While high concentrations of ESBL *Escherichia coli* were present, the organisms likely responsible for the urine discoloration, *Providencia stuartii* and *Proteus mirabilis*, were much less abundant. There is a disconnect between the provider’s ESBL-based ertapenem antibiotic selection and the actual bacteria likely responsible for the purple urine that prompted treatment. Another significant nuance to this case is the hospice setting in which it occurred. While treatment of symptomatic UTIs can be a warranted palliative measure, interventions directed at asymptomatic conditions are generally contradictory to principles of comfort care. Recurring painful IM injections, exposure to unnecessary broad-spectrum antibiotics, and disruption of the patient’s environment by placing them in isolation are all misaligned with the hospice philosophy of alleviating discomfort at the end of life. These contradictions exemplify how the visually salient findings of PUBS may lead to treatment decisions discordant with the underlying pathophysiology and clinical presentation. The overall fundamental tension in this case and in the literature is whether PUBS is an interesting benign finding meriting conservative cautiousness or an important herald of UTIs justifying the liberal use of empiric antibiotics to avoid sepsis and resulting morbidity and mortality. A cursory review of the available literature revealed a predominance of individual case reports and small case series, with a paucity of large observational or controlled studies, and most authors advocating early antibiotic treatment due to PUBS's reported association with UTIs. This seeming contradiction with our conventional understanding of asymptomatic bacteriuria, recommendations to avoid antimicrobials in that setting, and the principle of antibiotic stewardship, was the impetus for this review.

In addition to the index case report, we performed a structured aggregation of recently published case reports and case series in PUBS to descriptively examine symptom status, management patterns, and outcomes; this analysis was intended to be hypothesis-generating rather than a formal systematic review.

A literature review was conducted to identify case reports and case series describing PUBS. PubMed and PubMed Central were searched using combinations of the terms “purple urine bag syndrome, “PUBS, “purple urine, and “urinary catheter discoloration.” References cited within relevant articles were also reviewed to identify additional cases. The cases were included if sufficient clinical data were available to extract patient demographics, clinical presentation, microbiologic data, and management. Only case reports and case series published within the last 10 years were included to reflect contemporary diagnostic and management practices. Reports lacking sufficient information to assess symptom status or treatment were excluded.

The literature search was conducted through December 2025. Titles and abstracts were screened for relevance, and full-text review was conducted when available. Only full case reports or case series were included; review articles, conference abstracts, and reports without original patient-level data were excluded. Non-English articles were included when sufficient clinical detail could be extracted from the published report or translated text. When multiple cases were reported within a single publication, each patient was treated as a distinct case. When duplicate reports for the same patient were suspected, the most complete report was retained. Screening and data extraction were performed by a single reviewer. A total of 63 cases met the inclusion criteria and were aggregated for analysis [12-67].

For each included case, the following variables were extracted when reported: age, sex, comorbidities, presence of an indwelling urinary catheter, reported urinary or systemic symptoms, urinalysis findings, urine culture results, antibiotic administration, and catheter management. Data were abstracted from published reports and entered into a standardized data set. Findings were only included when explicitly reported. A lack of documentation for a variable was treated as missing data rather than a negative finding. Symptom status was assigned only when presence or absence was explicitly stated; cases with insufficient information were excluded from symptom-based comparisons.

Risk factors were recorded based on explicit reporting within each case and included advanced age, female sex, chronic catheterization, constipation, immobility or institutionalization, CKD, diabetes mellitus, and structural urologic abnormalities.

To minimize interpretive bias introduced by the author's conclusions, cases were reclassified using symptom-based criteria aligned with the IDSA guidance [9] for UTI diagnosis. Symptomatic UTI was defined by the presence of at least one accepted urinary or systemic feature, including fever, dysuria, suprapubic pain, flank pain, costovertebral angle tenderness, rigors, sepsis, or hemodynamic instability attributed to infection [9]. Cases lacking these features were classified as asymptomatic bacteriuria, regardless of urinalysis abnormalities or positive urine culture results. Findings such as a change in mental status, urine discoloration, malodor, pyuria, or bacteriuria alone were not considered sufficient to define symptomatic infection.

Descriptive statistics were used to summarize patient characteristics, risk factors, and management patterns. Categorical variables are presented as counts and percentages. Antibiotic use was compared between symptomatic and asymptomatic cases as an exploratory analysis. A risk ratio was calculated to descriptively assess the association between symptom status and antibiotic administration. Given the small sample size and the observational, case-based nature of the data, Fisher’s exact test was used for exploratory evaluation of associations between categorical variables. Statistical testing was performed for descriptive purposes only and was not intended to support causal inference or formal hypothesis testing. When reported, p-values were calculated using a two-tailed threshold of 0.05 for consistency with conventional reporting, but were interpreted descriptively rather than as evidence of statistical significance. No multivariable analysis was performed. No proprietary tools, scoring systems, or licensed instruments were used in this study. Case classification was performed using free publicly available clinical guideline definitions, including the IDSA clinical guidance criteria on symptomatic UTI [9]. 

A total of 63 published cases of PUBS met the inclusion criteria and were included in our analysis [12-67]. Extracted variables included patient demographics, symptomatology, microbiology, treatment decisions, catheter management, and reported outcomes. A summary of key clinical and laboratory characteristics as well as management patterns across the cohort is presented (Table 3) [12-67].

**Table 3:** Clinical characteristics, diagnostic findings, and management of reported purple urine bag syndrome *Broad-spectrum or intravenous therapy included carbapenems, fluoroquinolones, third or fourth-generation cephalosporins, or other intravenous beta-lactams, as reported by original authors [12-67]. Detailed case-level extracted variables are provided in Appendix A.

Variable	n (%)
Meets IDSA Criteria [9] for Symptomatic UTI	15 (24%)
Asymptomatic Bacteriuria	48 (76%)
Abnormal Urinalysis Reported	63 (100%)
Positive Urine Culture Reported	59 (94%)
Antibiotics Administered (Any)	52 (83%)
Antibiotics Given in Asymptomatic Patients	37 (77%)
Broad-Spectrum or Intravenous Therapy*	15 (24%)
Narrow-Spectrum Oral Therapy	37 (59%)
No Antibiotics Administered	11 (17%)
Urinary Device Exchanged or Removed	40 (63%)
Resolution of Purple Discoloration Reported	55 (87%)

Patients were predominantly elderly, female, chronically catheterized, and with frequent comorbidities, including immobility, constipation, CKD, and diabetes mellitus. The distribution of established PUBS risk factors in our cohort closely mirrored numbers reported in prior narrative reviews and case series (Table 4) [1-2,5,12-67].

**Table 4:** Risk factors associated with purple urine bag syndrome: comparison of the present cohort with previously reported literature *Approximate ranges derived from aggregated case reports and narrative reviews; precise denominators and age cutoffs are inconsistently reported [1-2,5,15,17,31,36,42,55,65]. Detailed case-level extracted variables are provided in Appendix B.

Risk Factor	Present Cohort (n = 63)	Previously Reported Literature*
Indwelling Urinary Catheter	56 (89%)	~85-100%
Age ≥ 70 Years	50 (79%)	~75-90%
Female Sex	49 (78%)	~70-90%
Bedridden/Institutionalized	41 (65%)	~60-80%
Constipation	39 (62%)	~50-70%
Chronic Kidney Disease (CKD)	25 (40%)	~30-50%
Diabetes Mellitus	19 (30%)	~25-40%
Structural Urologic Abnormality	9 (14%)	<20%

The concordance between risk factors observed in the present cohort and those described in prior reviews supports the representativeness and external validity of the aggregated dataset.

Using symptom-based criteria aligned with IDSA guidance [9], only 15 of 63 cases (24%) met criteria for symptomatic UTI. The remaining 48 cases (76%) lacked accepted urinary or systemic symptoms, despite almost universal abnormalities on urinalysis and cultures, and were therefore classified as asymptomatic bacteriuria. Urine cultures were obtained and reported in 59 cases (94%) and frequently demonstrated polymicrobial growth. Commonly isolated organisms included *Escherichia coli*, *Proteus species*, *Providencia *species, *Klebsiella* species, and *Morganella morganii* [10-11]. In many cases, organisms classically associated with PUBS were reported at low abundance alongside higher-burden organisms not typically implicated in pigment formation, mirroring the findings in our index case. ESBL-producing organisms were identified in a subset of *Escherichia coli* isolates, but did not consistently correlate with symptom status or adverse clinical outcomes.

Despite the predominance of asymptomatic presentations, 52 patients (83%) received systemic antibiotic therapy. Among patients classified as asymptomatic, 37 of 48 (77%) were managed with antibiotics. All symptomatic patients received antimicrobials. Antibiotic exposure included broad-spectrum or intravenous therapy in 15 cases (24%), narrow-spectrum oral therapy in 37 cases (59%), and no antibiotics in 11 cases (17%). Urinary device exchange or removal was reported in 40 cases (63%).

When antibiotic use was compared between symptomatic and asymptomatic cases, antibiotic prescribing was descriptively higher among symptomatic cases (risk ratio 1.30). However, antibiotic use remained common among asymptomatic cases. All comparisons should be interpreted as exploratory and descriptive, given the case-report-based nature of the dataset (Fisher’s exact test p = 0.053).

Resolution of purple discoloration was reported in 55 cases (87%). While the preponderance of cases reported favorable outcomes independent of antibiotic administration, three mortalities occurred in the context of established septic shock and critical illness. These three cases were classified as symptomatic and were appropriately managed with broad-spectrum antibiotics. Importantly, PUBS itself was not implicated as the cause of sepsis, nor did asymptomatic cases progress to systemic infection. These reports highlight the risk of conflating correlation with causation, as PUBS commonly occurs in chronically ill populations who are already at elevated baseline risk for adverse outcomes.

**Figure 2:**
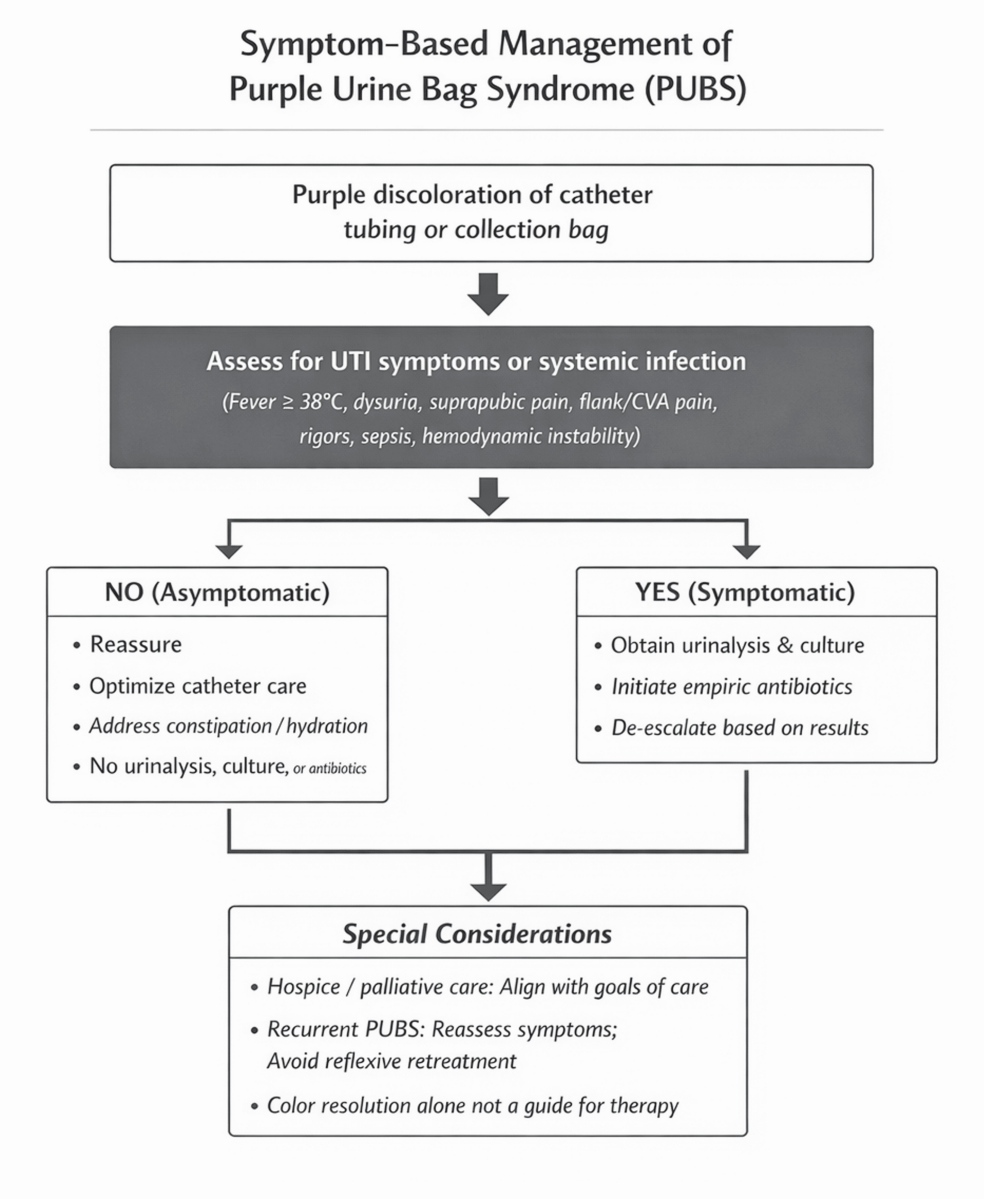
Symptom-based algorithm for the management of purple urine bag syndrome

In this aggregated analysis, most PUBS cases were asymptomatic, yet antibiotic therapy was frequently administered, including in patients who did not meet symptom-based criteria for UTI. This pattern was consistent across diverse reports and suggests that PUBS often functions as a visual trigger for antimicrobial treatment, rather than a reliable indicator of symptomatic infection. These observations define a recurring disconnect between common physician practice and guideline-based principles for the management of asymptomatic bacteriuria. A pervasive narrative throughout PUBS literature is that PUBS is a marker of UTI and a potential harbinger of severe infection and mortality, dictating prompt, and sometimes universal, antibiotic treatment. These conclusions are often supported by bacteriuria, abnormal urinalysis findings, and apparent resolution of urine discoloration following antibiotic administration. However, bacteriuria and pyuria are prevalent in populations most affected by PUBS and, in isolation, do not establish pathologic infection outside the context of symptomatology [9]. Our analysis challenges the assertion that PUBS warrants indiscriminate antimicrobial treatment and suggests that prior interpretations have conflated association with causation. While PUBS almost ubiquitously coexists with pyuria and bacteriuria, it does not reliably distinguish symptomatic infection from colonization and should not spur injudicious or imprudent antibiotic use. To translate these observations into a practical, stewardship-aligned framework, a symptom-based algorithmic approach to the evaluation and management of PUBS is summarized (Figure 2).

The reach of these findings extends beyond PUBS and reflects a broader, concerning misconception of the general urinary tract bacteriuria-infection spectrum. Antibiotic use in asymptomatic bacteriuria represents a prevalent mismanagement practice where treatment is often initiated in response to urine appearance, odor, laboratory findings, or culture results rather than patient symptoms [9]. PUBS serves as an especially vivid example of this phenomenon, with its dramatic purple appearance amplifying diagnostic urgency, overriding symptom-based reasoning, and prompting reflexive treatment. The downstream consequences of unnecessary antibiotic use, especially broad-spectrum or intravenous agents, are well established and include antimicrobial resistance, adverse drug reactions, drug-drug interactions, disruption of normal microbiota, and *Clostridioides difficile* infection, adversely affecting individual patient and public health outcomes [9]. Treating asymptomatic bacteriuria, whether prompted by PUBS or nonspecific findings, disregards the principles of antibiotic stewardship and exposes patients and their communities to harm without clear benefit. Recognizing PUBS as a component of the more overarching challenge of asymptomatic bacteriuria management may help reduce unnecessary antibiotic use and promote more patient-centered, stewardship-aligned care.

This study has several limitations. The analysis is based on published case reports and case series, which are subject to reporting bias and variable documentation. Publication bias is likely, as clinically dramatic or treated cases may be more frequently reported than conservatively managed cases. Symptom status may have been underreported in some cases, and management decisions were influenced by contextual factors not uniformly described. Additionally, some observations were derived from case series, and individual cases cannot be considered statistically independent. Also, the observational nature of the data and limited sample size restrict formal causal inference. Limiting inclusion of cases to the past decade may have excluded earlier reports; however, this approach was chosen to align findings with modern antibiotic stewardship principles and current clinical guidelines. Outcome data were inconsistently reported across cases, limiting conclusions regarding clinical progression or long-term effects. Despite these limitations, the consistency of findings across reports and alignment with established principles of UTI diagnosis and management support the conclusions.

## Conclusions

PUBS can be a visually alarming clinical phenomenon commonly occurring in patients with indwelling urinary devices and bacteriuria. In this aggregated analysis of published cases, PUBS was most frequently observed in the absence of accepted UTI symptoms, yet antibiotic therapy, often broad-spectrum, was commonly utilized. Our investigation is intended to challenge the prevailing narrative that PUBS should be viewed as an indicator of infection and precursor of severe illness requiring expedient antimicrobial treatment. Rather than functioning as an independent marker of pathology, PUBS more likely reflects underlying colonization in high-risk populations. As with other asymptomatic bacteriuria cases, PUBS therapy should be guided by symptom-based criteria, not urine color, urinalysis abnormalities, or culture results alone. Recognizing PUBS as an uncommon but illustrative component of the larger urinary tract bacteriuria-infection spectrum may promote more patient-centered, evidence-based, stewardship-aligned care.

## Appendices

Appendix A 

**Table 5:** Case-level clinical, microbiologic, and management data for reported purple urine bag syndrome cases Cases labeled Case 1, Case 2, etc., originate from published case series. IDSA, Infectious Disease Society of America; UA, urinalysis

First Author	Meets IDSA Symptomatic UTI Criteria	UA Result	Culture Result	Antibiotics Given	Antibiotic Class	Urinary Device Changed	Purple Urine Resolved
Shirsat [12]	False	Alkaline, Leukocyte Esterase, Nitrites,	*Proteus mirabilis*, *Klebsiella oxytoca*	True	Aminoglycoside	Yes	Yes
Oliveira [13]	True	Pyuria	Klebsiella oxytoca	True	Cephalosporin	Not Applicable	Yes
Kumar Case 1 [14]	False	Not Performed	Not performed	True	Nitrofuran	No	Yes
Kumar Case 2 [14]	False	Not Performed	Not performed	True	Nitrofuran	No	Yes
Hokama [15]	False	Nitrites, Hematuria, Pyuria	*Providencia stuartii*, *Pseudomonas aeruginosa*	False	-	Yes	Yes
Alex [16]	False	Alkaline, Hematuria, Pyuria	*Klebsiella pneumonia*, *Morganella morganii*, *Enterococcus*, *Citrobacter diversus*, *Pseudomonas aeruginosa*	False	-	Yes	Yes
Kenzaka [17]	False	Nitrites, Pyuria	Escherichia coli	True	Cephalosporin	Not Applicable	Yes
Mondragón-Cardona [18]	False	Alkaline, Nitrites, Pyuria	*Escherichia coli*, *Proteus mirabilis*, *Enterococcus faecalis*	True	Fluoroquinolone, Aminoglycoside	Yes	Yes
Van Keer Case 1 [19]	False	Alkaline, Pyuria, Hematuria	*Pseudomonas aeruginosa*, *Enterococcus faecalis*	False	-	Not Applicable	Yes
Van Keer Case 2 [19]	False	Alkaline, Pyuria, Hematuria	Escherichia coli	True	Penicillin	Yes	Yes
Mohamed [20]	False	Alkaline, Leukocyte Esterase, Hematuria	Mixed growth	True	Cephalosporin	Not Applicable	Yes
Abubacker [21]	False	Pyuria	Providencia stuartii	True	Cephalosporin	Yes	Yes
Karim [22]	False	Alkaline, Leukocyte Esterase, Nitrites, Pyuria	*Pseudomonas aeruginosa*, *Staphylococcus epidermidis*	True	Cephalosporin	Yes	Yes
Sriramnaveen [23]	False	Alkaline, Pyuria,	Pseudomonas aeruginosa	True	Cephalosporin	Not Applicable	Yes
Faridi [24]	True	Alkaline, Pyuria	Escherichia coli	True	Cephalosporin	No	Yes
Tul Llah [25]	True	Alkaline, Leukocyte Esterase, Nitrites, Pyuria, Hematuria	Proteus vulgaris	True	Cephalosporin, Sulfonamide	Not Applicable	Yes
Demelo-Rodríguez [26]	False	Alkaline, Nitrites, Pyuria	Klebsiella pneumoniae	True	Fluoroquinolone	Yes	Yes
Richardson-May [27]	False	Alkaline	Gram-negative coliforms	False	-	Yes	Not Reported
García-López [28]	False	Alkaline	*Citrobacter freundii*, *Enterococcus*	True	Cephalosporin	No	Yes
Koçoğlu [29]	False	Alkaline, Leukocyte Esterase, Pyuria, Hematuria	ESBL *Escherichia coli*	True	Cephalosporin, Carbapenem	No	Yes
Ficher [30]	False	Not Reported	Streptococcus agalactiae	True	Penicillin, Carbapenem, Glycopeptide	No	Not Reported
Agbor [31]	True	Alkaline, Leukocyte Esterase, Nitrites	Escherichia coli	True	Penicillin	Yes	Yes
Golzarri [32]	False	Pyuria, Nitrites	Klebsiella pneumoniae	True	Fluoroquinolone	Yes	Yes
Lin Case 1 [33]	True	Alkaline, Leukocyte Esterase, Pyuria	Vancomycin-resistant *Enterococcus*, *Proteus mirabilis*, *Escherichia coli*	True	Penicillin, Carbapenem, Cyclic Lipopeptide, Fluoroquinolone	Not Applicable	Yes
Lin Case 2 [33]	False	Alkaline, Nitrites	*Proteus mirabilis*, *Pseudomonas aeruginosa*, *Enterococcus*, *Alcaligenes* species	True	Fluoroquinolone	Not Applicable	Yes
Lin Case 3 [33]	False	Alkaline, Nitrites	Escherichia coli	False		Not Applicable	Yes
Le Mouel [34]	True	Alkaline, Pyuria	Klebsiella pneumoniae	True	Unspecified	No	Yes
Traynor [35]	False	Alkaline, Nitrites, Pyuria, Hematuria	Mixed growth	True	Nitrofuran	Yes	Yes
Karray [36]	True	Not Reported	Escherichia coli	True	Cephalosporin, Aminoglycoside	Yes	Yes
Pandey [37]	False	Alkaline, Pyuria	Escherichia coli	True	Unspecified	Yes	Not Reported
Worku [38]	False	Alkaline, Pyuria	*Escherichia coli*, Mixed growth	False	-	Yes	Yes
Shin Case 1 [39]	False	Alkaline, Nitrites, Hematuria	Not performed	False	-	Yes	Yes
Shin Case 2 [39]	False	Not Performed	Not performed	False	-	Yes	No
Boentoro [40]	True	Alkaline, Nitrites, Leukocyte Esterase	Escherichia coli	True	Cephalosporin, Fluoroquinolone	Yes	Yes
Carmo [41]	False	Pyuria, Hematuria	Nitrofuran-resistant *Proteus mirabilis*	True	Sulfonamide, Fluoroquinolone	Yes	Yes
Ong [42]	False	Not Reported	*Providencia rettgeri*,* Klebsiella pneumoniae*	False		Yes	Yes
Kumar [43]	False	Alkaline, Pyuria	Escherichia coli	True	Fluoroquinolone	Yes	Yes
Vetter [44]	True	Pyuria	Proteus mirabilis	True	Penicillin	Yes	Yes
Saraireh [45]	False	Alkaline, Pyuria, Hematuria	Proteus mirabilis	True	Fluoroquinolone	Yes	Yes
Kumar [46]	True	Alkaline, Leukocyte Esterase, Nitrites	Escherichia coli	True	Cephalosporin	Yes	Yes
Popoola [47]	True	Not Reported	Proteus species	True	Fluoroquinolone	Yes	Yes
Ramadan [48]	False	Alkaline, Leukocyte Esterase	Proteus mirabilis	True	Carbapenem, Nitrofuran	Yes	Yes
Ahmed [49]	True	Alkaline, Nitrites, Pyuria, Hematuria	Escherichia coli	True	Cephalosporin	Yes	Not Reported
Agbonghae [50]	False	Leukocyte Esterase, Pyuria	*Proteus mirabilis*,* Klebsiella pneumoniae*	True	Cephalosporin	Not Applicable	Yes
Nandwani [51]	False	Alkaline, Pyuria, Hematuria	Klebsiella pneumoniae	True	Unspecified	Yes	Yes
DeRon [52]	False	Leukocyte Esterase, Nitrites, Pyuria	*Proteus mirabilis*, *Escherichia coli*	True	Cephalosporin	Yes	Yes
Kannan [53]	False	Alkaline, Leukocyte Esterase, Nitrites, Hematuria	Proteus mirabilis	True	Cephalosporin	No	Not Reported
Tirtayasa [54]	False	Pyuria	*Escherichia coli*, *Pseudomonas aeruginosa*, *Klebsiella pneumoniae*	True	Carbapenem	No	Yes
Pallath [55]	False	Not Reported	*Escherichia coli*,* Proteus mirabilis*, Mixed growth	True	Unspecified	No	Not Reported
Popović [56]	False	Nitrites, Pyuria	Proteus mirabilis	True	Phosphonic	Yes	Yes
Jappi [57]	False	Alkaline, Pyuria	Enterococcus faecalis	True	Penicillin	Yes	Yes
Vanumu [58]	True	Alkaline, Leukocyte Esterase, Nitrites, Pyuria	*Escherichia coli*,* Klebsiella species*	True	Cephalosporin, Fluoroquinolone	Not Applicable	Yes
Faia [59]	False	Alkaline, Leukocyte Esterase, Nitrites, Pyuria	Providencia stuartii	True	Penicillin, Cephalosporin	Yes	Not Reported
Wong [60]	False	Alkaline, Pyuria, Hematuria	Mixed growth	True	Cephalosporin	Yes	Yes
Wang [61]	False	Alkaline, Pyuria	Escherichia coli	False	-	Yes	Yes
Pereira [62]	False	Alkaline, Nitrites, Pyuria	Morganella morganii	True	Sulfonamide	Not Applicable	Yes
Murray [63]	False	Leukocyte Esterase, Pyuria, Hematuria	Escherichia coli	True	Sulfonamide, Cephalosporin	No	Yes
Mahdi [64]	True	Nitrites, Pyuria	Klebsiella pneumoniae	True	Carbapenem, Glycopeptide	Yes	Yes
Joseph Case 1 [65]	False	Not Reported	*Pseudomonas aeruginosa*,* Proteus vulgaris*,* Citrobacter diversus*	True	Penicillin, Fluoroquinolone	Yes	Yes
Joseph Case 2 [65]	False	Not Reported	*Proteus mirabilis*,* Enterococcus*	True	Penicillin, Nitrofuran	Yes	Yes
Joseph Case 3 [65]	False	Not Reported	*Escherichia coli*,* Klebsiella pneumoniae*	True	Penicillin, Nitrofuran	Yes	Yes
Agrawal [66]	True	Alkaline	Escherichia coli	True	Aminoglycoside, Penicillin	Yes	Yes
Helmsdal [67]	False	Alkaline, Leukocyte Esterase, Nitrites	Escherichia coli	False	-	Yes	Yes

Appendix B 

**Table 6:** Case-level risk factor distribution for reported purple urine bag syndrome cases Cases labeled Case 1, Case 2, etc., originate from published case series.

First Author	Patient Age	Female Sex	Age ≥70	Foley Catheter	Bedridden/Immobile	Constipation	CKD	Diabetes Mellitus	Structural Urologic Abnormality
Shirsat [12]	70	True	True	True	False	True	True	True	False
Oliveira [13]	9	True	False	False	False	True	False	False	True
Kumar Case 1 [14]	56	True	False	True	True	True	False	False	False
Kumar Case 2 [14]	75	True	True	True	True	False	False	False	False
Hokama [15]	52	False	False	True	False	True	False	False	True
Alex [16]	83	False	True	True	False	False	True	False	False
Kenzaka [17]	72	True	True	False	False	False	False	False	True
Mondragón-Cardona [18]	71	True	True	True	True	False	False	False	False
Van Keer Case 1 [19]	81	False	True	False	False	True	True	False	False
Van Keer Case 2 [19]	80	False	True	True	False	False	True	True	False
Mohamed [20]	68	False	False	False	True	False	False	False	False
Abubacker [21]	36	False	False	True	False	True	False	False	False
Karim [22]	83	False	True	True	False	True	False	False	True
Sriramnaveen [23]	85	False	True	False	False	False	True	False	False
Faridi [24]	76	False	True	True	False	False	False	False	True
Tul Llah [25]	52	False	False	False	True	True	False	False	False
Demelo-Rodríguez [26]	83	False	True	True	False	False	False	True	False
Richardson-May [27]	94	True	True	True	False	True	False	False	False
García-López [28]	90	True	True	True	False	False	True	True	False
Koçoğlu [29]	78	False	True	True	False	False	True	False	False
Ficher [30]	83	True	True	True	False	False	True	False	False
Agbor [31]	80	True	True	True	True	True	False	False	False
Golzarri [32]	57	False	False	True	True	True	False	False	False
Lin Case 1 [33]	27	True	False	False	False	True	False	False	False
Lin Case 2 [33]	27	True	False	False	False	True	False	False	False
Lin Case 3 [33]	27	True	False	False	False	True	False	False	False
Le Mouel [34]	86	True	True	True	True	True	False	False	False
Traynor [35]	90	True	True	True	False	False	False	False	False
Karray [36]	78	False	True	True	True	False	False	True	True
Pandey [37]	70	False	True	True	False	True	False	False	False
Worku [38]	94	True	True	True	False	False	False	False	False
Shin Case 1 [39]	81	True	True	True	True	True	False	True	False
Shin Case 2 [39]	86	True	True	True	True	False	False	False	False
Boentoro [40]	64	True	False	True	False	True	False	False	False
Carmo [41]	65	False	False	True	False	False	False	False	True
Ong [42]	50	False	False	True	False	False	False	True	True
Kumar [43]	60	False	False	True	True	False	False	False	False
Vetter [44]	96	True	True	True	False	True	False	False	False
Saraireh [45]	80	True	True	True	False	True	True	True	False
Kumar [46]	52	True	False	True	False	True	False	False	False
Popoola [47]	90	True	True	True	False	True	False	False	False
Ramadan [48]	77	False	True	True	False	True	True	True	True
Ahmed [49]	74	True	True	True	True	False	False	True	False
Agbonghae [50]	56	False	False	False	False	False	False	False	False
Nandwani [51]	74	False	True	True	True	False	True	True	False
DeRon [52]	79	True	True	True	False	False	False	False	False
Kannan [53]	76	True	True	True	False	True	True	False	False
Tirtayasa [54]	27	True	False	True	False	False	False	False	False
Pallath [55]	70	True	True	True	False	False	False	False	False
Popović [56]	79	True	True	True	True	True	False	False	False
Jappi [57]	23	False	False	True	False	True	False	False	False
Vanumu [58]	73	True	True	False	False	True	True	False	False
Faia [59]	87	True	True	True	True	False	False	False	False
Wong [60]	86	True	True	True	False	False	False	False	False
Wang [61]	101	True	True	True	False	True	False	False	False
Pereira [62]	81	True	True	False	False	True	False	False	True
Murray [63]	46	True	False	True	False	False	False	False	False
Mahdi [64]	73	True	True	True	False	False	False	False	False
Joseph Case 1 [65]	50	False	False	True	True	False	False	False	False
Joseph Case 2 [65]	30	True	False	True	True	True	False	False	False
Joseph Case 3 [65]	70	True	True	True	False	False	False	False	False
Agrawal [66]	66	True	False	True	True	True	False	False	False
Helmsdal [67]	81	False	True	True	False	False	True	True	True
